# Modulation of Wheat Yield Components in Response to Management Intensification to Reduce Yield Gaps

**DOI:** 10.3389/fpls.2022.772232

**Published:** 2022-05-02

**Authors:** Brent R. Jaenisch, Lucas B. Munaro, S. V. Krishna Jagadish, Romulo P. Lollato

**Affiliations:** Department of Agronomy, Kansas State University, Manhattan, KS, United States

**Keywords:** intensive management, *Triticum aestivum* L., crop density, fungicide, fertility, biomass, kernels m^–2^, kernel weight

## Abstract

Appropriate genotype selection and management can impact wheat (*Triticum aestivum* L.) yield in dryland environments, but their impact on yield components and their role in yield modulation are not well understood. Our objectives were to evaluate the yield response of commercial winter wheat genotypes to different management practices reflecting a stepwise increase in management intensity (including a reduction in crop density under high input), and to quantify how the different yield components modulate wheat yield. A factorial experiment evaluated six management (M) intensities [“farmer practice” (FP), “enhanced fertility” (EF), “ecological intensification” (EI), “increased foliar protection” (IFP), “water-limited yield” (Yw), and “increased plant productivity” (IPP)] and four winter wheat genotypes (G) in four Kansas environments (E). Average grain yield was 4.9 Mg ha^–1^ and ranged from 2.0 to 7.4 Mg ha^–1^, with significant two-way interactions (E × M and E × G). The EF usually maximized yields in dry environments, while EI, which consisted of EF plus one fungicide application, maximized yields in environments with greater water availability. Across all sources of variation, kernels m^–2^ and aboveground biomass were the strongest modulators of yield as compared to kernel weight and harvest index, while spikes m^–2^ and kernels spike^–1^ modulated yields at a similar magnitude. Foliar fungicides improved yield through increased green canopy cover duration and greater radiation intercepted during grain filling. When crop density was reduced from 2.7 to 1.1 million plants per hectare in an otherwise high-input system, plants produced more productive tillers (with genotype-specific response); however, reduced green canopy cover at anthesis and reduced cumulative solar radiation intercepted during grain filling limited wheat yield—although large differences in canopy cover or intercepted radiation were needed to cause modest changes in yield. Treatments more intensive than EI were not warranted as EF or EI maximized yields at all environments, and practices that promote biomass and kernels m^–2^ are to be targeted for future increases in wheat yield.

## Introduction

Bread wheat (*Triticum aestivum* L.) is cultivated in more than 200 million ha across the world, being an essential component of the human diet and the primary source of calories for the world’s population (Reynolds et al., 2012). Thus, increases in wheat production are crucial for global food security (Shiferaw et al., 2013), especially as yield gains fail to sustain historical rates (Grassini et al., 2013). Within this context, increasing crop yield in current cropland can help to meet future food demand and minimize the expansion of agricultural lands (Cassman, 1999).

The majority of global wheat production occurs under rainfed conditions. These non-irrigated cropping systems are subject to droughts due to insufficient and/or poorly distributed precipitation (Sadras, 2002; Sadras and Angus, 2006; Torres et al., 2013; Lollato et al., 2017, 2020a). This leads to a more conservative approach from producers in terms of adoption of management practices with the objective of increasing yield. The underlying rationale is that water availability is the most yield-limiting factor and reduces the return on added inputs (Jaenisch et al., 2019; de Oliveira Silva et al., 2020b), following Liebig’s law of the minimum, which states that the growth of a plant is proportional to the scarcest of the essential nutrients available. However, empirical and theoretical evidence supports that crop yield might not be limited by a single factor but rather determined by interactions between two or more factors (Sadras, 2004; Cossani and Sadras, 2018; Carciochi et al., 2020). Thus, it can be hypothesized that improvements in crop management could increase grain yield despite water limitation (de Oliveira Silva et al., 2020b).

The state of Kansas (United States) provides a great case study for testing the management and genotype opportunities for future yield increases in dryland wheat-growing regions. With 3–4 million ha of winter wheat sown annually and a production of ∼8 million metric tons, Kansas is the largest winter wheat-producing state in the country (USDA-NASS, 2017). The crop is grown predominantly under dryland conditions (∼94%, USDA-NASS, 2018), with a 10-year average yield of 2.8 Mg ha^–1^, which corresponds to only 50–55% of the dryland yield potential (∼5.2 Mg ha^–1^; Patrignani et al., 2014; Lollato et al., 2017). A range of genotypic traits and agronomic management practices is proposed to modulate wheat yield in this region (Lollato et al., 2020b; Munaro et al., 2020; Jaenisch et al., 2021). For instance, improved fertility management, including the adoption of in-furrow starter fertilizer (McConnell et al., 1986; Lollato et al., 2013; Maeoka et al., 2020), increased nitrogen rates (Thomason et al., 2002; Walsh et al., 2018; Lollato et al., 2019a,^2021^), and use micronutrients (Zain et al., 2015), has been associated positively with yields. Likewise, genetic resistance to major diseases and its interaction with foliar fungicides are management variables of interest (Lollato et al., 2019b; de Oliveira Silva et al., 2020b; Cruppe et al., 2021). The role of crop density seems a variable and dependent resource availability (Fischer et al., 2019; Lollato et al., 2019b; Bastos et al., 2020); thus, its potential to interact with other practices (e.g., Jaenisch et al., 2019) deserves further exploration.

The studies above provided insights into individual management practices to improve wheat grain yield. Others attempted to quantify wheat yield response to intensified management, combining the prophylactic use of a number of inputs to minimize yield gaps (Mohamed et al., 1990; Jaenisch et al., 2019; Quinn and Steinke, 2019; de Oliveira Silva et al., 2020b; Herrera et al., 2020; Roth et al., 2021; Steinke et al., 2021). However, with few exceptions (de Oliveira Silva et al., 2020b,^2021^), these efforts mostly overlooked the mechanisms behind the yield responses and simply quantified the magnitude of yield improvements. Because organogenesis is linked to crop developmental stages (Slafer et al., 2021), we argue that it is relevant to maximize yield within the time frame of yield component determination.

The relationships between wheat yield and its components [i.e., biomass, harvest index (HI), spikes m^–2^, kernels spike^–1^, kernels m^–2^, and kernel weight] have been researched for decades across a wide range of environments (Austin et al., 1980, 1989; Calderini et al., 1999; Acreche et al., 2008; Slafer et al., 2014). The majority of the literature suggests that wheat is mostly sink limited, with kernels m^–2^ explaining a larger variation of yield than kernel weight, and with changes in assimilate supply only offering modest changes in yield (Slafer and Savin, 1994; Borrás et al., 2004; Slafer et al., 2014; and citations therein). Thus, management practices that affect kernels m^–2^ would expectedly have a greater impact on yield. Still, some management practices that mostly modulate kernel weight might also relate positively to yield in some environments (Cruppe et al., 2021). To our knowledge, there have been no attempts to explicitly manipulate management practices that match important stages of crop development when different organs are produced and quantify their relationship to yield within a context of management intensification, which is crucial for food security (Cassman and Grassini, 2020).

Organs that eventually become a source and a sink are initiated during different times in the vegetative and reproductive stages in wheat (Slafer and Rawson, 1994; Ochagavía et al., 2021). Crop density is determined during the vegetative stage as seedlings emerge and establish; tillers m^–2^ (and thus potential spikes m^–2^) are determined between seedling emergence and the terminal spikelet stage (although less productive tillers can be produced later); potential spikelets spike^–1^ is determined prior to the first visible node; and kernels spikelet^–1^ is determined between the onset of stem elongation until harvest maturity through the process of floret development (which ends by anthesis) and grain filling (Ochagavía et al., 2021). Grain weight is determined between booting and maturity, with different sensitivities to weather conditions between the heading and grain-setting stages (Calderini et al., 2001) as compared to and the grain filling stage (Bergkamp et al., 2018). Meanwhile, the source capacity (e.g., leaf area index) is usually maximized prior to anthesis and decreases with maturity (Lollato and Edwards, 2015). Disentangling the effects of genotype (G), environment (E), management (M), and their interactions—with the specific goal of modulating different yield components and tradeoffs—can provide a physiological basis for future yield increases in wheat.

While genotypic and management factors associated with wheat yield gaps in Kansas and other dryland regions have been explored individually in different studies, their role to improve crop yield and its components within an integrated management perspective having a goal to optimize yield components has not been explored. Thus, our objectives were to (i) evaluate the yield and yield components response of commercial winter wheat genotypes to different management practices reflecting a stepwise increase in management intensity using as baseline the current technology level followed by an average producer in the region and investigating levels of yield gaps; and (ii) quantify how different yield components modulate wheat yield in this dryland region. Because wheat response to crop density seems to depend on resource availability (Fischer et al., 2019; Bastos et al., 2020), we also tested whether reducing seeding rates from the most intensive treatment would be a promising strategy to reduce yield gaps. We hypothesize that a more intensive management will increase grain yield, and that yield increases will be genotype and environment specific. Additionally, we hypothesize that fertilizer-based practices will affect yield components that are coarse regulators of yield (i.e., spikes m^–2^ and kernels m^–2^), while fungicide-based practices will affect fine regulators of yield (i.e., kernel weight, kernels spike^–1^) (Slafer et al., 2014).

## Materials and Methods

### Experimental Locations and Agronomic Management

Rainfed field experiments were conducted in Kansas, United States, near Belleville (39.81°N, 97.67°W; 471 m; moderately well-drained Crete silt loam) and near Hutchinson (37.93°N, 98.03°W; 468 m; well-drained Ost loam) during the winter wheat seasons of 2017–2018 and 2018–2019. Each environment will be referred to as Bel18, Bel19, Hut18, and Hut19. Winter wheat was sown under conventional tillage after a summer fallow using a Great Plains 606 no-till drill (7 rows spaced at 19 cm) with plot dimensions of 1.3 m × 9.1 m. Seeds were treated with 6.9-g a.i. ha^–1^ thiamethoxam, 1.4-g a.i. ha^–1^ mefenoxam, and 8.9-g a.i. ha^–1^ difenoconazole to avoid early-season diseases and insects. Composite soil samples (i.e., 15 individual soil cores) were collected at sowing from the 0–15- and 15–60-cm depth to quantify initial soil nutrient status (Supplementary Table 1). Weeds were controlled and insect pressure was not observed across the study.

### Treatment Structure and Experimental Design

Treatments were arranged in a complete factorial structure established in a split-plot design with four replications. Whole plots were assigned to six management intensities, and sub-plots were assigned to four winter wheat genotypes. Treatment combinations represented stepwise increases in management intensity from a baseline reflecting the level of technology adoption of an average producer in the region and will, hereafter, be referred to as “farmer practice” (FP), “enhanced fertility” (EF), “ecological intensification” (EI), “increased foliar protection” (IFP), “water-limited yield” (Yw), and “increased plant productivity” (IPP) (Table 1).

**TABLE 1:** Description of the six management intensities and four winter wheat genotypes evaluated in the current study.

	Management intensity							Genotype			
Input	FP	EF	EI	IFP	Yw	IPP	Trait	WB4303	WB4458	WB-Grainfield	Zenda
N Rate for Yield Goal (Mg ha^–1^)	2.4	6.7	6.7	6.7	6.7	6.7	YOR	2017	2013	2012	2017
In-furrow starter N, P, S, and Zn	No	Yes	Yes	Yes	Yes	Yes	Maturity	ME	M to ME	M	M
Foliar Fungicide Feekes GS10.5	No	No	Yes	Yes	Yes	Yes	Straw strength	E	G	A	E
Foliar Fungicide Feekes GS6	No	No	No	Yes	Yes	Yes	Drought tolerance	BA	AA	AA	BA
Foliar S, Zn, Mg, and B	No	No	No	No	Yes	Yes	Stripe rust	MS	S	MR	MR
Seeding rate (million seeds ha^–1^)	2.7	2.7	2.7	2.7	2.7	1.1	Leaf rust	MS	S	MR	MR

*Farmer practice (FP) was followed by stepwise additions of five inputs: enhanced fertility (EF), ecological intensification (EI), increased foliar protection (IFP), water-limited yield potential (Yw), increased plant productivity (IPP). Abbreviations: YOR, year of release; M, medium maturity for heading date; ME, medium-early maturity for heading date; E, excellent straw strength; G, good straw strength; A, average straw strength; and for disease-resistant ratings, S, susceptible; MS, moderately susceptible; MR, moderately resistant. We note that these resistance ratings reflected the study period, but some cultivars have lost their resistance since the study was conducted.*

The FP consisted of a seeding rate of 2.7 million seeds ha^–1^ plus an N application at Zadoks GS23-25 with a rate reflecting a yield goal of the 10-year wheat grain yield average in the county where the experiment was located (∼2.4–2.8 Mg ha^–1^). The first increase in intensity was the enhanced fertility (EF) treatment, which included 112 kg ha^–1^ micro essentials (MESZ; 13-kg N ha^–1^, 45-kg P ha^–1^, 11-kg S ha^–1^, and 1-kg Zn ha^–1^) placed in a furrow with the seed, and an increased N rate for a 6.7 Mg ha^–1^ yield goal applied at Zadoks GS23–25 in the spring (Table 1). The fertilizer treatments aimed at increasing tiller and biomass production. The N rate in this treatment was selected so that N was not a limiting factor based on the long-term wheat yield potential of ∼5.2 Mg ha^–1^ (Lollato et al., 2017). The next step was ecological intensification (EI), which consisted of EF plus one fungicide application (fluxapyroxad-26 g ha^–1^, pyraclostrobin-171 g ha^–1^, propiconazole-107 g ha^–1^) at Zadoks GS55. Increased foliar protection (IFP) was the next step, consisting of EI plus the same fungicide product and the rate applied at Zadoks GS31. The aim of these fungicide applications was to protect the green canopy cover of the crop (i.e., source) during the different stages of development. The water-limited yield potential (Yw) treatment consisted of IFP plus micronutrients (81-g S ha^–1^, 90-g Zn ha^–1^, 67-g Mn ha^–1^, and 2-g B ha^–1^) applied at Zadoks GS31. Finally, the increased plant productivity (IPP) treatment was designed to explore whether a high resource availability scenario allowed for reduced crop density; thus, the seeding rate was 1.1 million seeds ha^–1^, reflecting the low seeding rates used by progressive growers in the region (Lollato et al., 2019b).

Wheat genotypes were selected based on their adoption by growers, adaptation to the region, and contrasting traits of interest for intensive management as well as performances in regional trials. The genotypes tested and their percent of the seeded area in central Kansas during 2020–2021 were WB4303 (<1%), WB4458 (2.2%), WB-Grainfield (5.5%), and Zenda (7.8%) (USDA-NASS, 2020). Information about traits of interest of each genotype within the context of management intensification is provided in Table 1.

The nitrogen rate was determined considering the soil NO_3_-N measured at sowing, potential N released from the organic matter, and a 40 kg ha^–1^-applied N per a Mg ha^–1^ grain yield goal (Leikam et al., 2003). Due to the residual soil NO_3_-N carry over from the previous growing season and estimated N release from organic matter, the N rate varied across environments (Supplementary Table 2). A pressurized CO_2_ backpack sprayer with a three-nozzle boom was used to apply the N as urea ammonium nitrate (UAN, 28-0-0) with a streamer nozzle (SJ3-03-VP), and foliar fungicide and micronutrients using a flat-fan nozzle (XR11002) with a constant volume of 140 L ha^–1^. Treatment application dates are provided in Supplementary Table 2.

### Measurements

Crop density was recorded in two linear meters per plot, 3–4 weeks after sowing. Percent green canopy cover was measured approximately at bi-weekly intervals from heading (Zadoks GS55) until maturity (Zadoks GS 95) from downward-facing digital photographs from an area of about 1 m^2^ processed using Canopeo (Patrignani and Ochsner, 2015). Aboveground biomass was sampled from a one-linear row-meter area (∼0.19 m^2^) from one of the center rows of each plot the same day of wheat harvest. Samples were dried at 65°C until constant weight and dry aboveground biomass were measured. The spikes were counted and separated from the stover prior to threshing to remove the chaff from the kernels. Grain weight was measured after threshing. The grain weight divided by the total aboveground biomass weight (including stover, chaff, and grain) determined the harvest index (HI). A 1,000-kernel weight was determined from a random kernel sub-sample. The ratio between total grain weight and 1,000 kernel weight determined kernels m^–2^; and the ratio between kernels m^–2^ by spikes m^–2^ determined kernels per spike. The number of productive tillers per plant was calculated as the ratio of spikes m^–2^ and plants m^–2^. Plots were trimmed prior to harvest to avoid edge effects, and wheat was harvested from a ∼13-m^2^ area using a small-plot Massey Ferguson 8XP combine. Grain moisture was measured at harvest, and grain yield was corrected for 135-g kg^–1^ water content. Grain protein concentration was measured using near-infrared spectroscopy.

Weather data, including precipitation, reference evapotranspiration (ETo), and maximum and minimum temperatures, were collected from a station pertaining to the Kansas Mesonet (Patrignani et al., 2020) located ∼50 m from the experiments. Plant available water at sowing was estimated using non-growing season precipitation and the soil’s available water-holding capacity (Lollato et al., 2016). At each environment, the weather variables were averaged (Tmax, Tmin) or accumulated (precipitation, ETo) for the entire growing season, as well as separated into four distinct phases: fall (the period between sowing and December 31); winter (January 1 to March 31), critical period [20 days prior to anthesis through 10 days afterward (Fischer, 1985)], and grain filling (10 days after anthesis through harvest). This sub-division intended to reflect (i) the conditions surrounding sowing that affect crop establishment and fall tiller initiation; (ii) the dormant period that can affect tillering and winterkill; and (iii) the yield determination period in the spring, similar to previous reports in the region (e.g., Lollato and Edwards, 2015).

### Statistical Analyses

Pearson’s correlation analysis was performed in R using the “corrplot” package (Wei et al., 2017) to determine the degree of linear association between the weather variables at the different periods and the measured crop variables. Because the data only derived from four environments, we relaxed the assumptions of *p*-values for this specific analysis to 0.15, while, for all other analyses, effects were significant at α = 0.05. ANOVA was performed using “lmerTest” in R software version 3.4.0 (Kuznetsova et al., 2017). Management, genotype, environment, and their interactions were fixed effects, while block nested within environment and management intensity nested within the block were random effects (the latter accounted for the split-plot design).

We used the stability method (Eberhart and Russell, 1966) to further understand the genotypic and management effects on grain yield and on productive tillers per plant (the latter to quantify the impact of crop density). This method consists of a linear regression of trait expression of each genotype (or management) versus an environmental index calculated as the mean trait expression of all genotypes at each environment minus the overall mean trait expression across all environments. Each management-by-environment combination was considered an environment (*n* = 24) for the genotype analyses (e.g., Ferrante et al., 2017; Lollato et al., 2021), and each genotype-by-environment combination was considered an environment (*n* = 16) for the management analyses (e.g., Raun et al., 1993). The slope (α) indicates whether the genotype has broad adaptability (α = 1) or adaptability specific to low (α < 1) or high- (α > 1) trait-expression environments, and is associated with phenotypic plasticity (Sadras and Richards, 2014). The intercept (β) is an estimate of the trait expression across environments; and a model goodness of fit index (i.e., R^2^) quantifies stability.

The modulators of yield in response to management were quantified as the relationships between yield components and yield using linear regression (e.g., de Oliveira Silva et al., 2020b). Differences in grain yield between the FP and each management for each genotype were calculated and regressed for: (i) all environment and management practices by wheat genotype combinations (*n* = 96), (ii) on average of each management intensity (*n* = 24; 6 managements × 4 environments), and (iii) on average for each genotype (*n* = 24; 6 managements × 4 genotypes). To understand the drivers of yield improvements in response to each step within the management intensification practices evaluated, we explored the relationships between the responsiveness of yield and the responsiveness of each yield component using linear regression (Slafer et al., 2014). Responsiveness was calculated as the ratio of each trait in a given management intensity over the same trait measured in the preceding management intensity so that we could quantify the effects of each management addition (e.g., responsiveness calculated as EF over FP associated with changes resulting from improved fertility).

Finally, we evaluated the green canopy cover data and the cumulative radiation intercepted during grain filling to better interpret the effects of fungicide and of crop density on grain yield in terms of source limitation. First, we calculated the linear slope of canopy cover dynamics between heading and maturity to detect whether the presence of foliar fungicides delayed canopy senescence, which would be indicated by a less-negative slope. This comparison was made between treatments EF and EI to isolate the effect of a single fungicide application at Zadoks GS55. Second, green canopy cover values at anthesis and their association with grain yield were compared for the Yw and IPP treatments to detect whether grain yield limitation from lower crop density could be explained by reduced green canopy cover. Finally, for the selected treatments above, cumulative radiation intercepted between anthesis and harvest maturity was calculated as the product between daily solar radiation and percent green canopy cover (Purcell, 2000). Daily values of green canopy cover were estimated for days between measurements using linear interpolation between consecutive measurements (Lollato and Edwards, 2015).

## Results

### Weather Conditions and Associations With Yield Components

**FIGURE 1:**
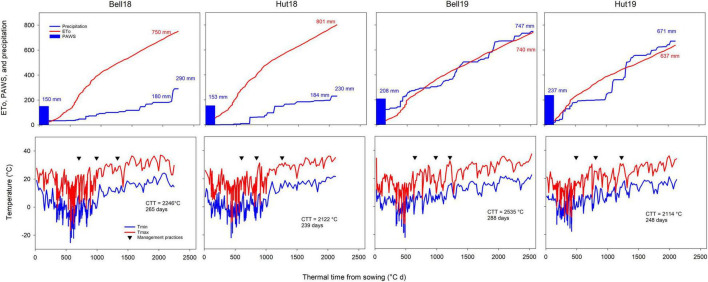
Weather conditions experienced during the winter wheat-growing season at the four Kansas environments resulting from two locations (Bell, Belleville; Hut, Hutchinson) and two growing seasons (18, 2017–2018 season; 19, 2018–2019 season). The upper row shows plant available water at sowing (PAWS), cumulative reference evapotranspiration (ETo) and precipitation, the bottom row shows maximum and minimum temperatures. Downward facing triangles show, respectively dates for N application at Zadoks GS25, fungicide and micronutrient application at GS32, and fungicide application at GS55. Inset values show cumulative ETo, precipitation, PAWS, cumulative thermal time between sowing and harvest (CTT), and season duration in days. Two cumulative precipitation values are shown for 2018 environments as considerable rainfall occurred after the crop was mature.

Growing season total precipitation ranged from 297 to 823 mm, and seasonal ETo ranged from 637 to 801 mm (Figure 1). Environments in 2017–2018 had a cold and dry fall, winter, and early spring, and a hot and dry late spring and early summer. Environments in 2018–2019 had warm and moist fall and cool and moist late spring and early summer, increasing disease pressure (Hollandbeck et al., 2019). Above-normal May and June temperatures in 2017–2018 (average temperatures between 23 and 27°C vs. 15–23°C in 2018–2019) accelerated and shortened the reproductive crop development (duration of grain fill, ranging from 27 to 29 days in 2017–2018 and from 33 to 52 days in 2018–2019; Figure 1), consequently decreasing the yield potential of the crop. The contrasting environments resulted in growing season length ranging from 239 to 288 days.

Table 2 shows the correlations between weather variables during specific crop developmental stages and yield components. Productive tillers plant^–1^ related negatively with fall Tmin and positively with Tmin during the critical period. Harvest index related positively to winter Tmin. Spikes m^–2^ related negatively to Tmin and precipitation during the winter. The negative relation between winter Tmin and spikes m^–2^ or productive tillers plant^–1^ reflects a delayed incorporation of the N fertilizer into the root zone until late spring in these environments, reducing the formation of spring tillers. Kernels spike^–1^ related positively to precipitation and water supply during the season, fall and grain-filling precipitation, and duration of the grain-filling period; and negatively to Tmax (growing season, and at each stage evaluated), and Tmin during grain filling. Kernel weight associated positively with winter Tmin and precipitation, as well as critical period precipitation.

**TABLE 2:** Correlations between yield components, averaged across four varieties and six management intensities, and daily average or cumulative values of environmental factors during specific crop development periods.

Trait	Environmental factor	Period	*r*
Productive tillers plant^–1^	Tmin	Fall	–0.99
	Tmin	Critical period	0.89
Harvest index	Tmin	Winter	0.96
Spikesm^–2^	Tmin	Winter	–0.88
	Precipitation	Winter	–0.87
Kernels spike^–1^	Tmax	Growing season	–0.99
	Precipitation	Growing season	0.97
	Water supply	Growing season	0.96
	Tmax	Fall	–0.89
	Precipitation	Fall	0.96
	Tmax	Winter	–0.91
	Tmax	Critical period	–0.86
	Tmax	Grain filling	–0.87
	Tmin	Grain filling	–0.9
	Precipitation	Grain filling	0.91
	Duration	Grain filling	0.86
Kernels m^–2^	Tmax	Winter	–0.88
	Precipitation	Grain filling	0.9
Kernel weight	Tmin	Winter	0.9
	Precipitation	Winter	0.93
	Precipitation	Critical period	0.89

*Weather variables included in the analysis were minimum (Tmin,°C) and maximum (Tmax,°C) temperatures, cumulative precipitation (mm), plant available water at sowing (PAWS, mm), water supply (growing-season precipitation plus PAWS, mm), and photothermal quotient (MJ m^–2^ C^–1^). Developmental periods evaluated were the fall (from the sowing date until December 31), the winter (from January 1 until March 31), the critical period (20 days prior to until 10 days after anthesis), and the grain-filling period (from 10 days after anthesis until harvest).*

### Management and Genotype Effects on Grain Yield and Yield Components

**FIGURE 2:**
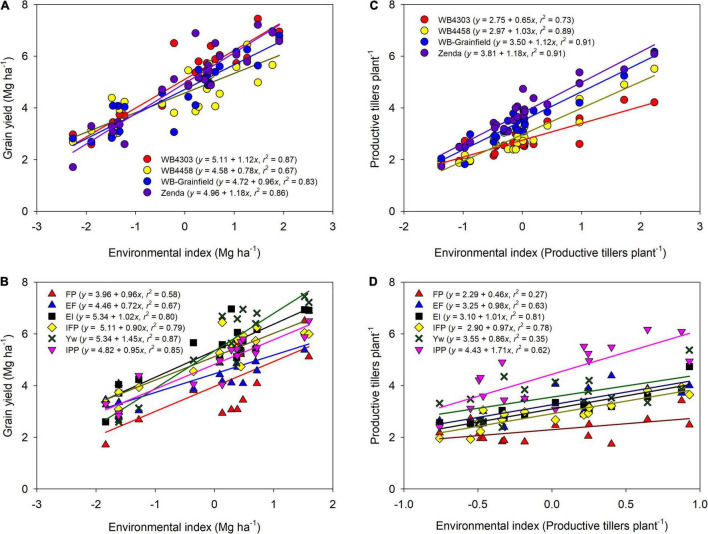
Wheat grain yield **(A,B)** and productive tillers per plant **(C,D)** as affected by the environment index for each wheat genotype (WB4303, WB4458, WB-Grainfield, and Zenda) **(A,C)** and management intensity (FP, farmer practice; EF, enhanced fertility; EI, ecological intensification; IFP, increased foliar protection, Yw, water-limited yield potential; and IPP, increased plant productivity) **(B,D).** Environmental indices were calculated as the combination of environment (Bel18, Hut18, Bel19, and Hut19) and **(A,C)** management practices or **(B,D)** genotypes.

Across all sources of variation, mean grain yield ranged from 2.3 to 7.2 Mg ha^–1^ (Figure 2). Environmental mean yield (across management and genotypes) ranged from 3.3 Mg ha^–1^ in Hut18 to 5.6 Mg ha^–1^ in Bel19, with overall greater yields in 2019 (5.43 Mg ha^–1^) as compared to 2018 (4.28 Mg ha^–1^). Mean grain yield for the genotypes was highest for WB4303 (5.11 Mg ha^–1^), followed by Zenda (4.96 Mg ha^–1^), WB-Grainfield (4.72 Mg ha^–1^), and WB4458 (4.58 Mg ha^–1^) (Figure 2A). Mean yield for the different management intensities was 3.96, 4.46, 5.34, 5.11, 5.34, and 4.82 for FP, EF, EI, IFP, Yw, and IPP, respectively (Figure 2B).

There were significant G × E and M × E interactions for grain yield, but no three-way interaction (Supplementary Table 3). General trends as related to the G × E interaction were: (i) WB4303 was in the highest yielding group at all environments; (ii) Zenda was in the highest yielding group in three out of four environments; and (iii) WB4458 yielded well in dryer conditions (i.e., Hut18) but yielded poorly at the higher-yielding environments (Bel19) (Table 3). General trends as related to M × E interaction were: (i) the FP yielded similarly to other treatments only in one environment (Bel18); (ii) EF yielded higher from FP in three environments; (iii) increases in grain yield from foliar protection (i.e., EI) only occurred in environments with greater rainfall (i.e., Bel19 and Hut19); (iv) the addition of the early fungicide (i.e., IFP) did not increase yields compared to a single fungicide application later in the season; (v) wheat grain yield benefited from all the management practices combined (i.e., Yw) only in one environment (i.e., Hut19); and (vi) reducing crop density under an otherwise highly managed system had no effect on grain yield except in one environment (i.e., Hut19) (Table 3).

**TABLE 3:** Least square mean winter wheat grain yield as affected by management practices (FP, EF, EI, IFP, Yw, and IPP), wheat genotypes (WB4303, WB4458, WB-Grainfield, and Zenda), and environments (Bel18, Hut18, Bel19, and Hut19).

	Environment				
	Bel18	Hut18	Bel19	Hut19	Mean

Genotype	Grain yield (Mg ha^–1^)				
WB4303	5.54Aa	3.17Bab	6.36Aa	5.32Aab	5.10
WB4458	5.29Aab	3.56Ba	4.49ABc	4.97Ab	4.58
WB-Grainfield	4.93Ab	3.22Bab	5.13Ab	5.56Aa	4.71
Zenda	5.22Bab	2.93Cb	6.44Aa	5.23Bab	4.96
Mean	5.25	3.22	5.61	5.27	
**Management**					
FP	5.31Aa	2.53Bb	4.62Ad	3.39Be	3.96
EF	4.91ABa	3.42Ba	5.06Acd	4.38ABd	4.44
EI	5.24Ba	3.60Ca	6.62Aa	5.89ABbc	5.34
IFP	5.29Aa	3.56Ba	5.55Abc	6.10Aab	5.13
Yw	5.37Ba	2.87Cab	6.32ABab	6.75Aa	5.33
IPP	5.34Aa	3.36Ba	5.46Abc	5.12Acd	4.82
Mean	5.24	3.22	5.61	5.27	

*Least square means followed by a common uppercase letter (comparisons across environments) or a lowercase letter (comparisons across management practices or genotypes) are not significantly different by the Tukey test at the 5% level of significance.*

Further exploration of the significant interactions through the adaptability and stability indices suggested that wheat genotypes varied in stability and adaptability across the different yield environments (Figure 2A). The wheat genotype WB4458 had the lowest α (0.78 ± 0.11), suggesting that this genotype was the least adapted to high-yielding environments and was unstable with a high variation about the fitted line (*R*^2^ = 0.67). Due to their α equal to one (1.12 ± 0.09, and 0.96 ± 0.09), the wheat genotypes WB4303 and WB-Grainfield showed broad adaptability and greater stability (*R*^2^ > 0.83), while Zenda was adapted to high-yielding environments (α = 1.18 ± 0.09). Management practices also showed environmental-specific adaptability, with EF showing greater yields in low-yielding environments (α = 0.72 ± 0.14), Yw showing adaptability to high-yielding environments (α = 1.45 ± 0.15), and the remaining management intensities showing broad adaptability (Figure 2B). Yield stability improved from the FP to the Yw treatments (*R*^2^ ranging from 0.67 to 0.87, Figure 2B).

With the exception of 1,000 kernel weight and grain protein, the yield components followed the yield analysis and were not affected by the three-way interaction, mostly reflecting G × E and M × E interactions (Supplementary Table 3). Briefly, in terms of crop density, the IPP treatment had fewer plants m^–2^ (149–163) as compared to other treatments (223–266 plants m^–2^) as expected (Table 4), which resulted in more productive tillers per plant (3.18–4.97 vs. 2.16–4.22) (Table 5). Management intensification tended to increase aboveground biomass as compared to the FP (magnitude: 18–100%), while the latter usually resulted in the greatest HI—with exception of Bel19 –although the magnitude of change was not large (16–46%) (Supplementary Table 4). The magnitude in the differences in spikes m^–2^ due to management and genotype was similar (38–72%) as those compared to changes in kernels spike^–1^ (39–64%) (Supplementary Table 5). The results of kernels m^–2^ reflected those for grain yield (Supplementary Table 6), while 1,000 kernel weight and grain protein were impacted by a G × E × M interaction (Supplementary Table 7).

**TABLE 4:** Least square mean winter wheat plants m^–2^ as affected by management practices (FP, EF, EI, IFP, Yw, and IPP), environments (Bel18, Hut18, Bel19, and Hut19) and genotypes.

	Environment					Genotype				
	Bel18	Hut18	Bel19	Hut19	Mean	WB4303	WB4458	WB-Grainfield	Zenda	Mean

Management	Plants m^–2^					Plants m^–2^				
FP	275.3Aa	231.3Aab	275.7Aa	196.0Ab	244.6	242.8Aa	245.0ABa	249.6ABa	240.9Aa	244.6
EF	284.1Aa	211.5Ab	264.9ABa	202.6Ab	240.8	236.9Ab	258.5ABa	226.1Bb	241.6Aab	240.8
EI	293.1Aa	224.2Ab	281.9Aa	207.0Ab	251.6	256.0Aa	254.2ABa	257.4Aa	238.6Aa	251.6
IFP	273.6Aa	233.1Aab	280.3Aa	203.7Ab	247.7	248.4Aab	266.0Aa	238.6ABb	237.6Ab	247.7
Yw	291.7Aa	219.2Ab	219.3Bb	195.0Ab	231.3	247.7Aa	228.1Bab	223.0Bb	226.5Ab	231.3
IPP	144.2Bb	100.7Bb	242.3ABa	138.5Bb	156.4	156.1Ba	157.6Ca	149.2Ca	162.8Ba	156.4
Mean	260.3	203.3	260.7	190.5		231.3	234.9	224.0	224.7	

*Least square means followed by a common uppercase letter (comparisons across management) or a lowercase letter (comparisons across environments or genotypes) are not significantly different by the Tukey test at the 5% level of significance.*

**TABLE 5:** Least square mean winter wheat productive tillers plants**^–^**^1^ affected by wheat genotypes (WB4303, WB4458, WB-Grainfield, and Zenda), environments (Bel18, Hut18, Bel19, and Hut19).

	Environment					Management						
	Bel18	Hut18	Bel19	Hut19	Mean	FP	EF	EI	IFP	Yw	IPP	Mean
		
Genotype	Productive tillers plant^–1^					Productive tillers plant^–1^						
WB4303	2.78Ca	2.73Ba	2.72Ba	2.99Ca	2.81	2.16Ac	2.66Bbc	2.54CBCc	2.50Bbc	3.16Cab	3.81Ba	2.81
WB4458	3.48Ba	2.93Bab	2.51Bb	2.94Cab	2.97	2.19Ac	2.70Bbc	2.90BCbc	2.60Bc	3.36Cab	4.06Ba	2.97
WB-Grainfield	3.91Aa	3.47Aab	3.08Ab	3.52Bab	3.50	2.26Ac	3.62Ab	3.11Bb	3.22Ab	3.79Bb	4.97Aa	3.50
Zenda	4.14Aa	3.62Aab	3.29Ab	4.20Aa	3.81	2.57Ac	4.0Abc	3.78Abc	3.37Ac	4.22Aab	4.90Aa	3.81
Mean	3.5775	3.1875	2.9	3.4125		2.295	3.2525	3.0825	2.9225	3.6325	4.435	

*Least square means followed by a common uppercase letter (comparisons across genotypes) or a lowercase letter (comparisons across environments or genotypes) are not significantly different by the Tukey test at the 5% level of significance.*

Different genotypes had different tillering abilities and adaptation to tillering environments, which were mostly modulated by reduced crop density (Figure 2C). Zenda had the highest tillering ability across environments (mean: 3.81 productive tillers per plant) with even greater tillering expression in high-tillering environments (α = 1.18 ± 0.19), which was followed by WB-Grainfield, WB4458, and WB4303 (3.50, 2.97, and 2.75 productive tillers per plant) (Figure 2C). While WB-Grainfield and WB4458 had wide adaptability of productive tillers per plant, the ability of WB4303 to produce tillers decreased in reference to the other genotypes as tillering environment increased (α = 0.66 ± 0.13). Reduced crop density (IPP) allowed for the greatest expression and maintenance of tillers (mean of 4.43 productive tillers plant^–1^), which increased at α = 1.71 ± 0.36 with the environmental index for tillering production (Figure 2D). The lowest tillering production and response to tillering environment occurred at the FP (mean of 2.29 productive tillers plant^–1^, α = 0.46 ± 0.20).

### Yield Component Modulation of Wheat Grain Yield

**FIGURE 3:**
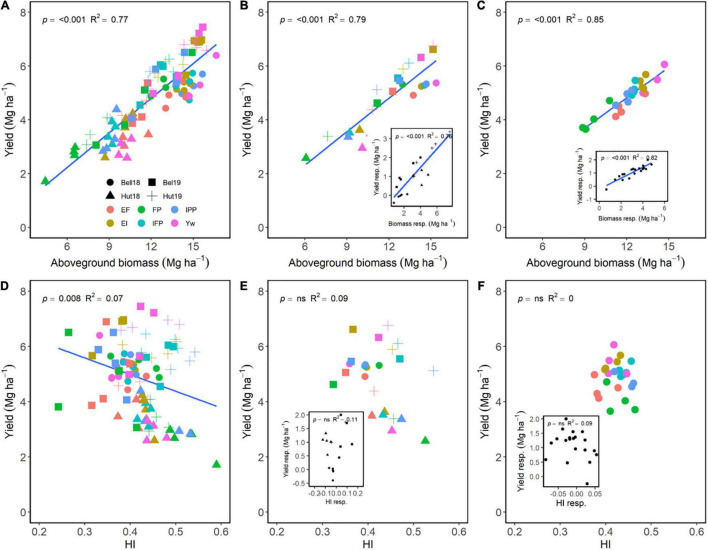
Relationship between yield and aboveground biomass **(A–C)** or harvest index **(D–F)** at maturity across environments, wheat genotypes, and management systems (*n* = 96) **(A,D)**, on average of each management for each environment (*n* = 24; 6 management practices × 4 environments) **(B,E)**, on average of each genotype for each environment (*n* = 24; 6 management practices × 4 genotypes) **(C,F)**. Inset graphs are the relationships between the responses of the variables to each management practices (difference between each management practice from the FP) averaged across either genotype for each management practice (*n* = 20) or management for each environment (*n* = 20) **(C,F)**.

Across E, M, and G, aboveground biomass at maturity explained 77% of the variation in yield, showing a positive relationship (Figure 3A). Although significant, a negative relationship of HI only explained 8% of the variation in yield (Figure 3D). Across environments, differences in grain yield were dependent on differences in biomass accumulation (Figure 3B) and independent of differences in HI (Figure 3E). Following the same trend, differences in biomass accumulation among the different wheat genotypes under different managements were also strongly related to differences in grain yield (Figure 3C) as compared to HI (Figure 3F). Increasing management intensity (the difference of each management practice to FP) significantly increased biomass accumulation, which increased yield across environments (Figure 3B, insert). Likewise, increased management intensity increased the responsiveness of biomass accumulation for wheat genotypes, which increased grain yield (Figure 3C, insert). Meanwhile, increased management intensity had limited effect on HI across environments or across genotypes (Figures 3E,F, inserts).

**FIGURE 4:**
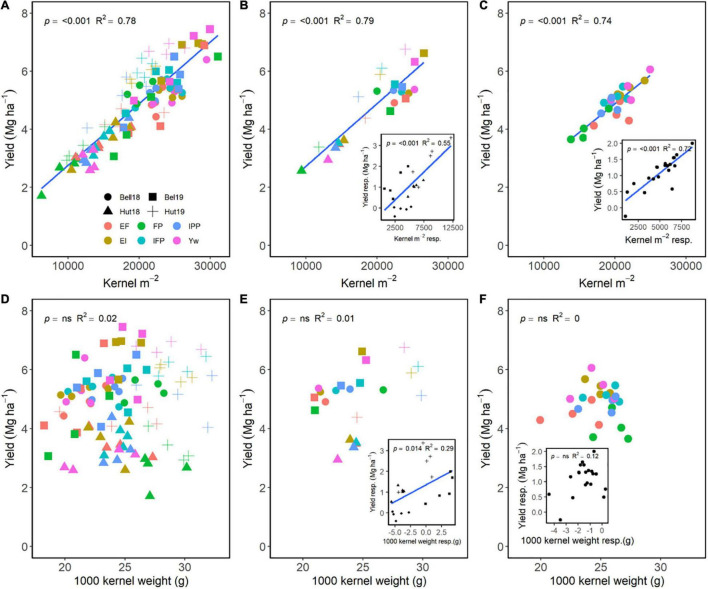
Relationship between yield and kernels m**^–^**^2^ **(A–C)** or 1,000 kernel weight **(D–F)** across environments, wheat genotypes, and management systems (*n* = 96) **(A,D)**, on average, each management for each environment (*n* = 24; 6 management practices × 4 environments) **(B,E)**, on each genotype for each environment (*n* = 24; 6 management practices × 4 genotypes) **(C,F)**. Inset graphs are the relationships between the responses of the variables to each management practices (difference between each management practice from the FP) averaged across either genotype for each management practice (*n* = 20) or management for each environment (*n* = 20) **(C,F)**.

Kernels m^–2^ had greater importance in increasing grain yield as compared to kernel weight (Figure 4). Across E, M, and G, a positive relationship of kernels m^–2^ explained 78% of the variation in grain yield (Figure 4A), while no relationship between kernel weight and yield occurred (Figure 4D). Averaged across genotypes, increasing management intensity increased grain yield through differences in kernels m^–2^ (Figure 4B), and yield responses to management practices were associated with increases in kernels m^–2^ (Figure 4B, insert). Similarly, averaged across management practices, wheat genotypes that had greater kernels m^–2^ also had greater grain yield (Figure 4C), and yield responses were dependent on the genotype’s kernels m^–2^ responsiveness (Figure 4C, insert). Following a different trend, increases in grain yield were independent of kernel weight for both management practices and wheat genotypes (Figures 4D–F); however, increases in kernel weight due to management were associated with increased grain yield within environment (Figure 4E, insert). Differences in kernel weight within each genotype were not associated with increases in grain yield (Figure 4F, insert).

**FIGURE 5:**
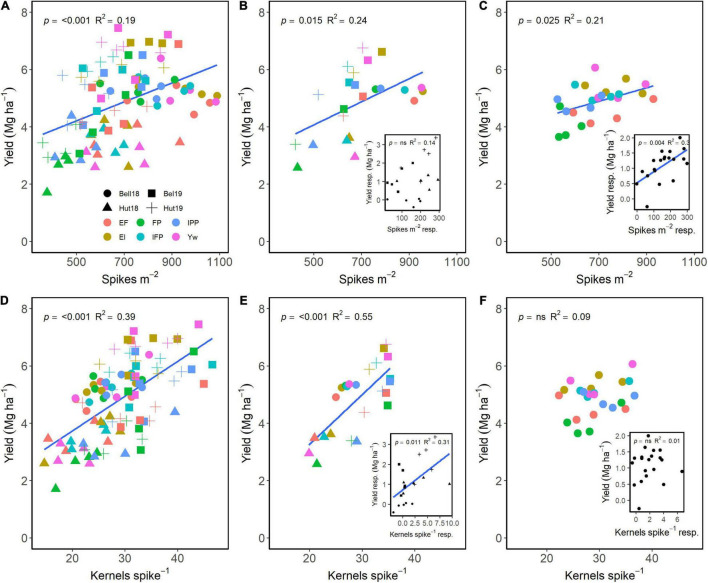
Relationship between yield and spikes m**^–^**^2^ **(A–C)** and kernels spike**^–^**^1^ **(D–F)** across environments, wheat genotypes, and management systems (*n* = 96) **(A,D)**, on average, each management for each environment (*n* = 24; 6 management practices × 4 environments) **(B,E)**, on each genotype for each environment (*n* = 24; 6 management practices × 4 genotypes) **(C,F)**. Inset graphs are the relationships between the responses of the variables to each management practice (difference between each management practice from the FP) averaged across either genotype for each management practice (*n* = 20) or management for each environment (*n* = 20) **(C,F)**.

Spikes m^–2^ and kernels spike^–1^ both had a positive effect on grain yield (Figure 5). Across G, E, and M, a positive relationship of spikes m^–2^ and of kernels spike^–1^ explained 19 and 39% of the variation in yield, respectively (Figure 5A). Averaged across either management practices or wheat genotypes, grain yield differences were dependent on differences in spikes m^–2^ (Figures 5B,C). Likewise, wheat genotype responsiveness to spikes m^–2^ resulted in positive differences in grain yield (Figure 5C, insert). Interestingly, management practices resulting in greater number of kernels spike^–1^ also significantly affected yield (Figure 5E), but there were no differences across genotypes (Figure 5F). Likewise, the responsiveness of kernels spike^–1^ to management practices affected grain yield, with no differences among genotypes (Figure 5F, inserts).

**FIGURE 6:**
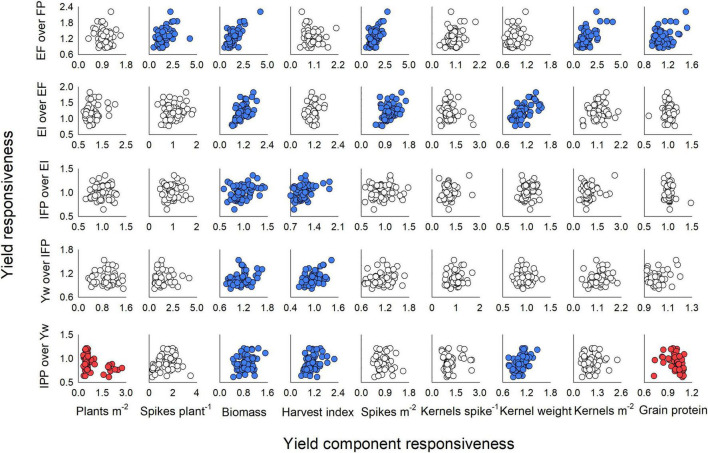
Winter wheat yield responsiveness and its relationship with responsiveness of yield components (plants m**^–^**^2^, biomass, harvest index, spikes m**^–^**^2^, kernels spike**^–^**^1^, and kernel weight) and grain protein concentration for each step of management intensification evaluated in the current study. Responsiveness values were calculated as enhanced fertility (EF) over farmer’s practice (FP) (first row); ecological intensification (EF) adding a fungicide application at Zadoks GS55 over EF (second row); increased foliar protection (IFP), adding a fungicide application at Zadoks GS31 to EI (third row); rainfed yield potential (Yw), adding micronutrients at Zadoks GS31 to the IFP (fourth row); and increased plant productivity (IPP), reducing the seeding rate from Yw (fifth row). Circles in blue denote a significant positive and circles in red a significant negative relationship between variables at *p* < 0.05.

Each stepwise increase in management intensity modulated different yield components (Figure 6). In the first step (i.e., addition of enhanced fertility to the FP), the responsiveness of yield ranged from 0.85 to 2.22 (mean: 1.23 ± 0.03) and was positively linked to the responsiveness of the productive tillers plant^–1^ (range: 0.48–4.28, mean: 1.49 ± 0.06), biomass (range: 0.50–4.26, mean: 1.40 ± 0.06), spikes m^–2^ (range: 0.53–2.75, mean: 1.40 ± 0.04), and kernels m^–2^ (range: 0.39–4.12, mean: 1.44 ± 0.06) (Figure 6, first row). We also note that yield responsiveness was positively associated with grain protein responsiveness (range: 0.93–1.52, mean: 1.11 ± 0.01) when fertility drove yield increase. When one fungicide application was added to the EF, yield responsiveness ranged from 0.77 to 1.82 (mean: 1.20 ± 0.02) and associated positively with responsiveness of biomass (range: 0.61–1.86, mean: 1.14 ± 0.03), spikes m^–2^ (range: 0.55–1.68, mean: 1.06 ± 0.02), and kernel weight (range: 0.79–1.58, mean: 1.11 ± 0.02) (Figure 6, second row). The addition of an early fungicide application to the EI had very weak relationships of yield responsiveness (range: 0.65–1.36, mean: 1.02 ± 0.01) to the responsiveness of biomass (range: 0.58–1.44, mean:0.97 ± 0.02) and HI (range: 0.70–1.84, mean: 1.08 ± 0.03) (Figure 6, third row). Likewise, the addition of micronutrients to the IFP treatment suggested that responsiveness of biomass (range: 0.77–1.67, mean: 1.12 ± 0.06) and of HI (range: 0.62–1.43, mean: 0.96 ± 0.03) associated with responsiveness of yield (range: 0.81–1.53, mean: 1.06 ± 0.01) (Figure 6, fourth row). Finally, when crop density was reduced from the Yw, responsiveness in yield (range: 0.61–1.21, mean: 0.91 ± 0.01) was positively related to responsiveness of biomass (range: 0.45–1.53, mean: 0.89 ± 0.03), of HI (range: 0.54–2.20, mean: 1.08 ± 0.03), and of kernel weight (range: 0.75–1.47, mean: 1.05 ± 0.02), and negatively related to responsiveness of plants m^–2^ (range: 0.32–2.75, mean: 0.80 ± 0.06) and protein (range: 0.72–1.10, mean: 1.00 ± 0.01) (Figure 6, fifth row).

**FIGURE 7:**
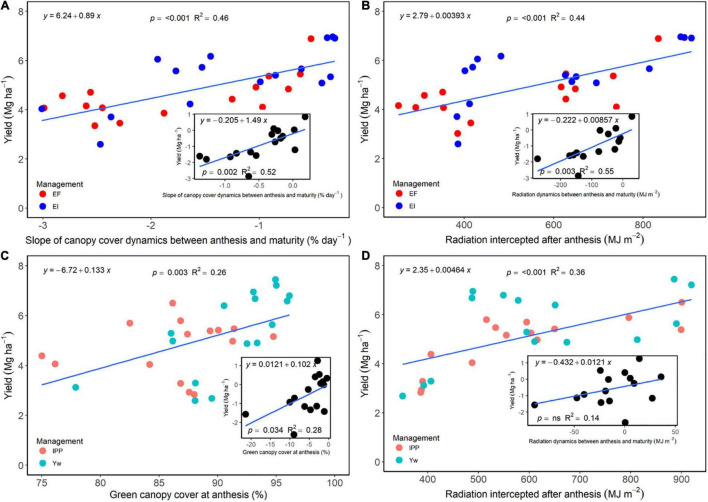
**(A)** Relationship between wheat grain yield and a slope of the green canopy cover dynamics between anthesis and maturity for the enhanced fertility (EF) and ecological intensification (EI) treatments across genotypes and environments. The inset panel in **(A)** shows the relationship between the difference in both grain yield and a canopy cover dynamics slope between the two treatments. **(B)** Relationship between wheat grain yield and percent green canopy cover values measured at anthesis for the “yield potential” (Yw) and “increased plant productivity” (IPP) treatments across genotypes and environments. The inset panel **(B)** shows the relationship between the difference between IPP and Yw for grain yield and percent green canopy cover. **(C)** Relationship between wheat grain yield and radiation dynamics between anthesis and maturity for the EF and EI treatments across genotypes and environments. The inset panel in **(C)** shows the relationship between the difference in both grain yield and radiation dynamics between the two treatments. **(D)** Relationship between wheat grain yield and radiation values measured at anthesis for the Yw and IPP treatments across genotypes and environments. The inset panel **(D)** shows the relationship between the difference between IPP and Yw for grain yield and radiation.

The slope of green canopy cover dynamics following fungicide application, as well as the cumulative radiation intercepted during the grain filling period, was positively associated with grain yield for the selected treatments that allowed for a direct comparison between fungicide and non-fungicide application (EF versus EI) (Figures 7A,B). Likewise, the difference between slopes of these treatments or intercepted radiation was positively related to grain yield difference (Figures 7A,B, insert). For each individual slope, intercept, and regression fit, please refer to Supplementary Table 8. Following a similar trend, green canopy cover values measured at anthesis, and the cumulative radiation intercepted after anthesis for the Yw and IPP treatments, related positively with grain yield (Figures 7C,D), as did their differences (Figures 7C,D, insert), providing empirical evidence for the reason behind decreased yields from reduced crop density in an otherwise well high-input system.

## Discussion

We aimed to expand on the knowledge of the interactions G × E × M to identify opportunities for future yield increases for dryland winter wheat through yield component manipulation using Kansas, United States, as a case study. The average grain yield in the FP was 4.01 Mg ha^–1^, which resulted in a yield gap of 1.37 Mg ha^–1^ when compared to the highest yielding treatment (Yw). Similar yield levels and yield gaps have been reported for the area under intensified management (Jaenisch et al., 2019, 2021; de Oliveira Silva et al., 2020b), confirming the opportunity to increase current yields through management intensification.

The management comprised of enhanced fertility plus one foliar fungicide application around heading (i.e., EI) resulted in average yield of 5.36 Mg ha^–1^, which was similar to the Yw treatment, although the latter received an additional fungicide application and micronutrients. Thus, these additional practices might not be necessary to fill the bulk of the yield gap, although this was environment-dependent (i.e., Hut19). Additionally, in environments where water deficit limited the yield potential of the crop, EF was sufficient to maximize grain yield, precluding application of foliar fungicides. Furthermore, in one dry environment with high NO_3_-N carryover (Bell18), the FP was enough to maximize grain yield. These findings support the idea that managing with the goal of reaching the yield potential might not be economical (Lobell et al., 2009).

Wheat genotypes responded differently to increased yielding conditions but similarly to management (Figure 2 and Table 3), suggesting that selecting wheat genotypes either with performance specific to the most reoccurring environment in a given region or with broad adaptability seems more promising than genotype-specific management. We note, however, that the lack of significant G × M interaction in this research might be due to a small sample size, as previous research with larger sample size showed significant G × M (Thompson et al., 2014; Cruppe et al., 2021).

### Management Practices and Their Effects on Wheat Yield Components

Our results align well with previous literature reporting that, across all sources of variation, wheat grain yield relates closely to aboveground biomass and kernels m^–2^, and is relatively independent of harvest index and kernel weight (Slafer et al., 2014; Ferrante et al., 2017; de Oliveira Silva et al., 2020b). However, an original contribution of our research is the detailed yield responsiveness analysis and its relation to yield component responsiveness for each individual step in management intensification (Figure 6). To our knowledge, this has not been previously attempted in the existing literature of wheat response to management intensification. From this analysis, it was clear that the yield responsiveness was greater for added fertility (EF) and one fungicide application (EI) (mean responsiveness of 1.20–1.23) as compared to the remaining practices (mean responsiveness of 0.91–1.06). The added fertility drove improvements in yield mostly through greater number of productive tillers plant^–1^, biomass, spikes m^–2^, and kernels m^–2^, while the added fungicide modulated yield through biomass, spikes m^–2^, and kernel weight (Figure 6). Interestingly, the reduced crop density mostly decreased yield (responsiveness: 0.91) through reductions in biomass (responsiveness: 0.89), although there was some compensation through increased in harvest index (responsiveness: 1.08). The remaining practices only slightly modulated biomass and harvest index, having little effect on yield.

The modulation of yield through kernels m^–2^ driven by the added fertilizer (EF) is justified as both in-furrow P fertilizer, and N fertilizer increases tiller initiation (Spiertz and De Vos, 1983; Rodríguez et al., 1999), and N fertilizer can reduce floret abortion (Ferrante et al., 2010; González et al., 2011). Tiller production determines the potential spikes m^–2^, and floret development determines the potential kernels spike^–1^. Both yield components interact with environmental conditions to determine kernels m^–2^, which were highly positively related to yield (Figure 4). Thus, N availability has to meet the requirements for both of these processes during the growing season as untimely N deficiency can result in floret abortion and reduce kernels m^–2^, potentially reducing yield. Nitrogen rates offer an opportunity for increased yields (Lollato et al., 2021), especially in favorable seasons where the crop can capitalize on a greater yield potential (Cruppe et al., 2017; Lollato et al., 2019a). Expected N uptake based on yield potential can serve as a guide for managing N rates in the season (Leikam et al., 2003); and, for wheat, a recent synthesis of global literature has suggested that N uptake ranges from ∼20 to 400 kg N ha^–1^ (de Oliveira Silva et al., 2020a). Thus, matching N availability with the time when the potential number of kernels m^–2^ is determined (i.e., early stem elongation) results in yield increases as grain number is the dominant driver of yield (Borrás et al., 2004; Slafer et al., 2014). We also note that this developmental stage coincides with the greatest N uptake rate by the crop, which increases under intensive management (de Oliveira Silva et al., 2021).

Kernels m^–2^ and kernel weight are affected by complex interactions among many environmental factors in the late reproductive stages. Our results support available literature that suggests that kernels m^–2^ is a coarse regulator of wheat yield as compared to kernel weight (Borrás et al., 2004; Slafer et al., 2014), which is justified as each individual kernel has a narrow range in size (Sadras, 2007); thus, greater increases in grain yield come from filling more kernels (Borrás et al., 2004). We note, however, that increases in kernel weight through management associated positively with increases in yield (insert, Figure 4E), in particular through the application of foliar fungicides (Figure 6). These findings agree with previous reports of highly managed wheat in the U.S. Great Plains (Lollato and Edwards, 2015; Jaenisch et al., 2019; Cruppe et al., 2021) suggesting that kernel weight might, in some conditions, partially explain increases in yield for wheat.

Foliar diseases can occur prior to anthesis and last throughout the grain-filling period, coinciding with a period of significant demand for photosynthesized resources by the developing grain (i.e., a very strong sink; Fischer, 1985). These foliar diseases decrease the green leaf area of the plant (Schierenbeck et al., 2019), reducing radiation interception and radiation use efficiency (Schierenbeck et al., 2016), and ultimately decreasing the source of assimilates to the developing sink. This mismatch between a reduced assimilate supply (i.e., source) during a period with large demand can cause kernel abortion and reduce yield (Ferrante et al., 2010; González et al., 2011). Foliar fungicides can also increase kernel weight under severe disease infestations, which can reflect increases in grain yield (Cruppe et al., 2021), although this increase is environment specific (Lynch et al., 2017). Wheat kernel weight is sensitive to environmental stresses (e.g., heat or drought) between booting to anthesis when carpel (which will turn into the external grain structures) growth increases rapidly (Calderini et al., 2001), and from anthesis to maturity during kernel weight determination (Bergkamp et al., 2018). Foliar diseases during these developmental stages can reduce kernel weight, which could reduce yield (Figure 4E, insert; Figure 6). Similarly, increases in kernel weight associate with kernel-filling rate, and foliar diseases can reduce the rate of fill due competition for assimilates (Simmons et al., 1982).

Foliar fungicides maintain the yield potential at time of application by protecting the upper canopy and spikes, which supply a large portion of the carbohydrates that determine yield (Rawson et al., 1983) and can increase kernels m^–2^ (Brinkman et al., 2014). The prolonged green leaf area maintained through fungicides also allows for longer duration of active photosynthesis, ultimately increasing N uptake (de Oliveira Silva et al., 2021) and grain yield (Joshi et al., 2019; Nehe et al., 2020). This was shown in the current research as a more negative slope of the green canopy cover dynamics and a lower cumulative radiation interception after anthesis in the treatments not receiving foliar fungicides (Figure 7). The positive relationship between the slope of canopy cover and grain yield also suggests that treatments not receiving foliar fungicides were, at least, to some extent, source limited, which was also evidenced by the greater grain protein concentration of treatments receiving foliar fungicides (data not shown). Further evidence for this source limitation is shown in the inset of Figures 4E, 6, in which increases in kernel weight through management associated positively with yield increases. However, we note that large reductions in the green leaf area or radiation intercepted were needed to cause modest reductions in yield (Figure 7), likely because wheat is mostly sink-limited and very efficient in translocating stem reserves to the developing kernels (Borrás et al., 2004). Even though foliar fungicides applied around anthesis have increased wheat yield and reduced the yield gap in the region (Thompson et al., 2014; Jaenisch et al., 2019), producers may be reluctant to apply it consistently due to high environmental unpredictability (Couëdel et al., 2021) and inconsistencies in yield response (Cruppe et al., 2021).

The evaluation of a reduced crop density under an otherwise highly managed system (IPP) suggested that yield responsiveness was negatively related to responsiveness in plants m^–2^ (Figure 6), reflected on the overall yield reduction of IPP as compared to Yw (4.82 vs. 5.39 Mg ha^–1^; Supplementary Table 3). This aligns with findings suggesting that crop density is an important determinant of the yield gap in rainfed wheat (Tokatlidis, 2014). Furthermore, it seems like the opportunity to reduce crop density in dryland conditions for winter wheat might not be as evident as that for irrigated spring wheat in low latitudes (Fischer et al., 2019), likely due to the unpredictability of conditions for tillering in the fall, which is dependent on many environmental variables (Tokatlidis, 2014). Nonetheless, we showed that there was a large genotypic component of tillering plasticity (Figure 2C) that might be further explored in this region. Tillering allows wheat plants to compensate for a low crop density, with greater opportunities in higher-yielding environments (Bastos et al., 2020), which was shown in this study with the IPP producing more tillers than other treatments. Tillering plasticity regulates the ability of a given genotype to tiller in different environments, which also interacts with crop density. Thus, a wheat variety with high-tillering potential and tillering plasticity (e.g., Zenda, Figure 2) has the ability to produce more productive tillers at reduced density (Figure 2C) and modulate yield through harvest index and kernel weight (Figure 6). On the other hand, a variety with low-tillering potential and plasticity (e.g., WB4303, Figure 2) is reliant on higher crop densities to attain desirable yields because individual plants are inefficient in using available resources (Tokatlidis, 2017). Evidence for other cereals suggests that high phenotypic plasticity of tillering can result in increased panicle weight under low-seeding rates (Kikuchi et al., 2017). Thus, selecting wheat genotypes for increased tillering capacity through conventional breeding could help reduce the risk associated with low-crop density (Fischer et al., 2019), which aligns with the early concept (Fasoulas, 1973) and more recent developments (Tokatlidis et al., 2006; Fasoulas, 2013) of selecting per-plant yield under nil competition.

### Genotypic Characteristics to Increase Grain Yield

Wheat genotypes responded to the environment differently but not to management practices or to the interaction of management and environment. Thus, our findings suggest that wheat genotypes have to be adapted to specific reoccurring environmental conditions or broadly adaptable and have other desirable agronomic traits, such as high-yield potential (Ferrante et al., 2017), disease resistance (Serrago et al., 2011), heat or drought stress tolerance (Bergkamp et al., 2018), to match those commonly experienced in the environment where the genotype is grown. While the lack of G × E × M in our data might result from the limited number of observations (i.e., four environments), previous research in the region also only found weak evidence for G × E × M in response to management intensification (*p* = 0.14; de Oliveira Silva et al., 2020b).

The wheat genotype WB4303 was better adapted to higher-yielding environments and responded to increased environmental index by producing more kernels m^–2^, which was highly correlated with increases in grain yield (Figure 4). These findings agree with those for other growing regions where modern genotypes were more adapted to higher-yielding environments and led to the hypothesis that the growers use older genotypes in their lowest-yielding soils and modern genotypes in their highest-yielding soils (Ferrante et al., 2017). While we did not test this hypothesis in Kansas, our findings suggest that this could be a promising strategy as the older genotype WB4458 was more adapted to lower-yielding environments, although further research is needed on this topic. For producers, selecting newer released genotypes might offer opportunities to capitalize on their ability to capture greater yields in higher-yielding environments (Slafer and Andrade, 1993; Perronne et al., 2017; de Oliveira Silva et al., 2020b) despite the challenge of finding information on new genotypes coupled with their limited life span (Perronne et al., 2017).

## Conclusion

The results from this research confirmed a large yield gap that can be fulfilled through management, while highlighting the opportunity to modulate different yield components through specific management practices in a stepwise increase in management intensification. Overall, the results reinforced the need for an integrated wheat management based on crop scouting, as environmental conditions determined which management practices resulted in the greatest grain yields; in higher-yielding, high-moisture environments, increased fertility and one application of foliar fungicide at anthesis seemed to maximize grain yields; while in lower-yielding, dry environments, increased fertility alone was sufficient to maximize grain yields—and the increased fertility was only warranted over farmer’s practice when the soil did not have enough fertility at sowing.

This research also confirmed the important role of aboveground biomass and kernels m^–2^ in maximizing grain yield at the expense of harvest index and kernel weight. Likewise, management of fertility led to yield modulation through improved biomass and kernels m^–2^. We note, however, that independent steps in management intensification impacted different yield components, and a fungicide application around Zadoks GS55 had an important impact on grain yield partially through biomass, kernel weight, and maintenance of green canopy cover longer into the grain-filling period. While the positive relation between green canopy cover (or radiation interception) during grain filling and yield suggests some potential for source limitation, large changes in green canopy cover were needed to cause modest changes in yield. The reduction of crop density in an otherwise highly managed system provided varying results and seems to limit yield through decreased green canopy cover at anthesis, decreased radiation interception during the grain-filling period, harvest index, and kernel weight. Thus, future research could focus on optimizing seeding rates and identifying genotypes with increased phenotypic plasticity of tillering to maximize winter wheat yields within a highly managed system.

## Data Availability Statement

The raw data supporting the conclusions of this article will be made available by the authors, without undue reservation.

## Author Contributions

BJ: conceptualization, data curation, formal analysis, data acquisition, investigation, methodology, and writing—review and editing. LM: statistical analysis and writing—review and editing. KJ: writing—review and editing. RL: conceptualization, data curation, formal analysis, funding acquisition, investigation, methodology, project administration, resources, software, supervision, visualization, and writing—review and editing. All authors contributed to the article and approved the submitted version.

## Conflict of Interest

The authors declare that the research was conducted in the absence of any commercial or financial relationships that could be construed as a potential conflict of interest.

## Publisher’s Note

All claims expressed in this article are solely those of the authors and do not necessarily represent those of their affiliated organizations, or those of the publisher, the editors and the reviewers. Any product that may be evaluated in this article, or claim that may be made by its manufacturer, is not guaranteed or endorsed by the publisher.
