# Supplementary material for: Modulation of Wheat Yield Components in Response to Management Intensification to Reduce Yield Gaps
Source: Front Plant Sci. 2022 May 2;13:772232. doi: 10.3389/fpls.2022.772232 (PMC9108894; doi:10.3389/fpls.2022.772232)
Supplement: Supplementary file 1 [file Data_Sheet_1.pdf]

SUPPLEMENTAL TABLE 1. Initial soil fertility at Belleville and Hutchinson, Kansas for the 2017-18 and 2018-19 growing seasons. Soil test includes soil pH, Mehlich-3 extractable phosphorus (P), potassium (K), calcium (Ca), magnesium (Mg), sodium (Na), ammonium-(NH_4-N) and nitrate- (NO_3-N) nitrogen, chloride (Cl), sulfate-sulfur (SO_4-S), organic matter (O.M.) and cation exchange capacity (C.E.C). Sampling depths were 0-15 cm and 15-60 cm.

Location	Year	Depth	pH	P	K	Ca	Mg	Na	NH_4-N	$N0_3$-N	Cl	$S0_4$-S	O.M.	C.E.C
		Cm					mg kg^{-1}						%	Meq 100g^{-1}
Belleville	2017-18	0-15	4.9	29	321	1465	204	13	2	20	2	3	2.7	25
		15-60	5.7	8	213	2450	300	28	2	14	2	2	2.5	23
Hutchinson	2017-18	0-15	6.0	77	218	1886	238	11	4	6	7	3	2.4	20
		15-60	6.7	55	214	2665	231	10	5	8	6	4	2.4	16
Belleville	2018-19	0-15	5.4	52	437	2056	296	17	3	1	8	3	3.1	28
		15-60	6.6	8	381	4022	555	58	5	4	9	3	2.4	26
Hutchinson	2018-19	0-15	8.0	27	315	4746	163	35	3	17	8	3	2.9	26
		15-60	8.1	4	194	5202	132	128	4	13	12	13	2.2	28

SUPPLEMENTAL TABLE 2. Sowing, harvest, and treatment application dates for the activities performed at Belleville and Hutchinson, Kansas, during the 2017-18 and 2018-19 growing seasons. Nitrogen rates applied at the different treatments are shown.

Year	Location	Sowing	N rate for FP (kg N ha^{-1})	N Rate for remaining treatments (kg N ha^{-1})	N application date	Micronutrients and fungicide at Feekes GS6	Fungicide at Feekes GS10.5	Harvest
2017-18	Belleville	10/2/2017	0	103	3/8/2018	4/27/2018	5/15/2018	6/23/2018
	Hutchinson	10/19/2017	10	170	3/2/2018	4/9/2018	5/9/2018	6/14/2018
2018-19	Belleville	10/3/2018	50	196	4/2/2019	4/29/2019	5/16/2019	7/17/2019
	Hutchinson	10/22/2018	0	110	3/18/2019	4/16/2019	5/15/2019	6/26/2019

SUPPLEMENTAL TABLE 3. Significance of the analysis of variance for environment (E), management (M), and genotype (G) effects on grain yield, grain protein concentration, aboveground biomass, and yield components (1000-kernel weight, spikes m^{-2}, and kernels m^{-2}).

Source of Variation	Yield	Protein	Plants m^{-2}	Aboveground biomass	Productive tillers $plant^{-1}$	Kernel m^{-2}	1000-Kernel weight	Spikes m^{-2}	Kernels $spike^{-1}$	HI
		---------- P > F ----------								
E	0.001	0.001	0.001	0.001	0.105	0.001	0.001	0.001	0.001	0.062
M	0.001	0.001	0.001	0.001	0.001	0.001	0.001	0.001	0.001	0.013
G	0.001	0.001	0.013	0.012	0.001	0.001	0.001	0.001	0.001	0.017
E×M	0.001	0.001	0.015	0.001	0.24	0.026	0.001	0.007	0.031	0.001
E×G	0.001	0.001	0.174	0.001	0.001	0.001	0.001	0.019	0.001	0.056
M×G	0.562	0.096	0.029	0.204	0.014	0.456	0.038	0.020	0.872	0.113
E×M×G	0.347	0.023	0.790	0.339	0.321	0.092	0.011	0.428	0.483	0.150

SUPPLEMENTARY TABLE 4. Least square mean winter wheat aboveground biomass and harvest index as affected by management practices (FP, EF, EI, IFP, Yw, and IPP), wheat genotypes (WB4303, WB4458, WB-Grainfield, and Zenda), and environments (Bel18, Hut18, Bel19, and Hut19). Least square means followed by a common uppercase letter (comparisons across environments) or lowercase letter (comparisons across management practices or genotypes) are not significantly different by the Tukey test at the 5% level of significance.

	Aboveground biomass					Harvest index				
Environ.	Bel18	Hut18	Bel19	Hut19	Mean	Bel18	Hut18	Bel19	Hut19	Mean
Genotype	----------- Mg ha^{-1} --------------					-------------unitless--------------				
WB4303	14.5Aab	9.2Ca	13.7ABab	11.6BCab	12.3	0.39Bab	0.46ABa	0.38Bab	0.48Aa	0.43
WB4458	14.7Aa	9.3Ba	11.5Bc	11.3Bb	11.7	0.40ABab	0.44Aa	0.36Bb	0.46Aab	0.42
WB-Grainfield	13.6Ab	9.1Ba	12.8Ab	12.1Aab	11.9	0.41Aa	0.47Aa	0.39Aa	0.48Aa	0.44
Zenda	14.3Aab	9.0Ba	14.2Aa	12.4Aa	12.5	0.37Ab	0.45Aa	0.40Aa	0.43Ab	0.41
Mean	14.3	9.2	13.1	11.9		0.39	0.46	0.38	0.46	
Management										
FP	13.0Ab	6.0Bb	11.2Ac	7.6Bd	9.5	0.42Ba	0.52Aa	0.32Cc	0.46ABb	0.43
EF	13.7Aab	10.6Ba	12.3ABc	10.8Bc	11.9	0.39Aab	0.41Ab	0.35Ac	0.41Ab	0.39
EI	14.3Aab	9.8Ba	15.2Aa	13.0Ab	13.1	0.40Aab	0.44Ab	0.37Abc	0.45Ab	0.42
IFP	14.5Aab	9.3Ba	12.6Abc	13.4Ab	12.5	0.39Aab	0.43Ab	0.47Aa	0.47Ab	0.44
Yw	15.4Aa	10.1Ba	14.3Aab	15.2Aa	13.8	0.36Ab	0.45Ab	0.42Aab	0.44Ab	0.42
IPP	14.7Aa	9.1Ca	12.8ABbc	11.1BCc	11.9	0.39BCab	0.47ABab	0.36Cbc	0.54Aa	0.44
Mean	14.3	9.2	13.1	11.9		0.39	0.45	0.38	0.46	

SUPPLEMENTARY TABLE 5. Least square mean winter wheat spikes m^{-2} and kernels spike^{-1} as affected by management practices (FP, EF, EI, IFP, Yw, and IPP), wheat genotypes (WB4303, WB4458, WB-Grainfield, and Zenda), and environments (Bel18, Hut18, Bel19, and Hut19). Least square means followed by a common uppercase letter (comparisons across environments) or lowercase letter (comparisons across management practices or genotypes) are not significantly different by the Tukey test at the 5% level of significance.

	Spikes m^{-2}					Kernels spike^{-1}				
Environ.	Bel18	Hut18	Bel19	Hut19	Mean	Bel18	Hut18	Bel19	Hut19	Mean
Genotype	---------------Heads m^{-2}---------------					----------- Kernels head^{-1} ------------				
WB4303	721Ac	522BCb	638ABc	498Cc	595	32.6Ca	26.6Da	43.6Aa	36.9Ba	34.9
WB4458	872Ab	544Bb	612Bc	547Bc	644	25.5Bb	25.7Ba	31.5Ab	29.6Ab	28.1
WB-Grainfield	911Ab	649Ba	721Bb	613Bb	724	26.1Bb	22.3Cb	33.0Ab	35.4Aa	29.2
Zenda	996Aa	678Ca	808Ba	729BCa	803	23.4Bb	17.7Cc	31.4Ab	25.3Bc	24.5
Mean	875	598	695	597		26.9	23.1	34.9	31.8	
Management										
FP	759Ab	430Cb	626Bc	423Cd	560	26.9Bab	21.3Cbc	34.9Aa	28.0Bc	27.8
EF	921Aa	711Ba	706Babc	624Bb	741	25.0Cb	21.2Cbc	34.6Aa	30.4Bbc	27.8
EI	957Aa	651Ca	785Ba	667BCab	765	26.1Bab	24.0Bb	34.1Aa	31.3Aabc	28.9
IFP	882Aa	634Ba	649Bbc	642Bab	702	27.1Bab	23.1Cbc	35.3Aa	32.7Aab	29.6
Yw	951Aa	666Ba	727Bab	705Ba	762	27.5Bab	20.0Cc	34.9Aa	34.5Aa	29.2
IPP	780Ab	497Bb	673Abc	518Bc	617	28.8Ba	29.0Ba	35.3Aa	33.8Aab	31.7
Mean	875	598	694	597		26.9	23.1	34.9	31.8	

SUPPLEMENTARY TABLE 6. Least square mean winter kernels per m^2 as affected by management practices (FP, EF, EI, IFP, Yw, and IPP), wheat genotypes (WB4303, WB4458, WB-Grainfield, and Zenda), and environments (Bel18, Hut18, Bel19, and Hut19). Least square means followed by a common uppercase letter (comparisons across environments) or lowercase letter (comparisons across management practices or genotypes) are not significantly different by the Tukey test at the 5% level of significance.

	Kernels m^{-2}				
Environ.	Bel18	Hut18	Bel19	Hut19	Mean
Genotype	--------------Kernels m^{-2}---------------				
WB4303	23609Aba	13465Ca	27547Aa	20296Bab	21229
WB4458	21999Aa	13830Ba	19171ABc	17959ABb	18240
WB-Grainfield	23505Aa	14544Ba	23822Ab	21860Aa	20933
Zenda	22850Aa	11878Ba	25312Aab	19877Aab	19979
Mean	22991	13429	23963	19998	
Management					
FP	20004Ab	9232Bb	21859Ab	17226Ab	17080
EF	22431Aab	14334Ba	24020Aab	22053Aa	20710
EI	24467Aa	15295Ba	26583Aa	22195Aa	22135
IFP	23459Aab	14736Ba	22504Ab	19876Aab	20144
Yw	25259Aa	12857Bab	25294Aab	20027Aab	20859
IPP	22324Aab	14121Ba	23518Aab	18612Aab	19644
Environmental mean	22991	13429	23963	19998	

SUPPLEMENTARY TABLE 7. Average wheat protein concentration and 1000 kernel weight as affected by management practices (FP, EF, EI, IFP, Yw, and IPP), wheat genotypes (WB4303, WB4458, WB-Grainfield, and Zenda), and environments (Bel18, Hut18, Bel19, and Hut19). For both variables, the three way interaction (G × E × M) was significant with Honest Significant Differences of 14.9 g kg-1 for grain protein concentration and 0.45 g for 1000 kernel weight.

		Grain protein concentration)						1000 Kernel weight					
Env.	Genotype	FP	EF	EI	IFP	Yw	IPP	FP	EF	EI	IFP	Yw	IPP
		-------------------- g kg^{-1} -----------------						------------------------- g ------------------------					
Bel18	WB4303	124	138	141	143	142	144	27.8	22.1	22.1	24	21.7	24.5
	WB4458	124	139	136	137	139	136	28.3	23.4	23.6	24.5	21.4	24.8
	WB-Grainfield	118	132	129	133	131	131	25	19.9	19.7	20.3	20.1	22.3
	Zenda	122	131	130	131	132	133	25.9	22.2	20.6	22.3	22.2	24.2
Hut18	WB4303	117	136	133	138	150	136	26.8	23.6	23	25.1	20.8	24.4
	WB4458	121	134	130	141	138	142	30.2	27.3	25.4	24.6	25.9	23.9
	WB-Grainfield	107	138	136	141	144	135	26.9	22	22	23.3	20	23.2
	Zenda	102	138	143	142	159	131	26.9	24.6	25	24.9	24.5	25.5
Bel19	WB4303	127	131	129	131	129	137	20.9	21.3	24.3	25.2	24.8	22.8
	WB4458	128	130	131	131	135	140	20.8	21	24.5	25.3	26.1	23
	WB-Grainfield	117	119	121	124	123	127	18.6	18.3	24.7	21.8	23.8	21
	Zenda	115	121	125	129	126	129	23.7	23.3	26.4	26.8	26.5	26
Hut19	WB4303	101	111	112	115	115	118	28.6	23.9	30.6	30.2	28.9	32.3
	WB4458	101	108	119	123	121	126	29.9	27.7	29.7	31.8	31.4	31.9
	WB-Grainfield	94	108	109	112	109	114	27	19.6	28	28.7	26	26
	Zenda	102	110	118	123	119	126	30.3	26.9	27.9	27.8	27.7	29.4

SUPPLEMENTARY TABLE 8. The relationships (intercept, slope, and R^2) between wheat grain yield and canopy cover slopes (pictures taken from anthesis to maturity) values affected by management practices (FP, EF, EI, IFP, Yw, and IPP), wheat genotypes (WB4303, WB4458, WB-Grainfield, and Zenda), and environments (Bel18, Hut18, Bel19, and Hut19).

Year	Management	Genotype	Belleville			Hutchinson		
			Intercept	Slope	R^2	Intercept	Slope	R^2
			Kg ha^{-1}	% day^{-1}		Kg ha^{-1}	% day^{-1}	
2018	FP	WB4303	9137	-0.51	0.29	46019	-2.6	0.99
		WB4458	4197	-0.23	0.09	35859	-2.03	0.91
		WB-Grainfield	13452	-0.76	0.29	58117	-3.29	0.99
		Zenda	6978	-0.39	0.18	48998	-2.77	0.89
	EF	WB4303	18359	-1.03	0.45	44657	-2.52	1
		WB4458	11018	-0.62	0.34	28554	-1.61	0.81
		WB-Grainfield	22164	-1.25	0.64	52959	-2.99	0.98
		Zenda	12931	-0.73	0.37	40619	-2.29	0.87
	EI	WB4303	15196	-0.85	0.44	41941	-2.37	0.77
		WB4458	6013	-0.34	0.2	29088	-1.64	0.81
		WB-Grainfield	17515	-0.99	0.68	53223	-3.01	0.85
		Zenda	7501	-0.42	0.45	43752	-2.47	0.85
	IFP	WB4303	7556	-0.42	0.44	51436	-2.91	0.93
		WB4458	5496	-0.31	0.18	40833	-2.31	0.9
		WB-Grainfield	12156	-0.68	0.57	57308	-3.24	0.96
		Zenda	4908	-0.27	0.44	49093	-2.77	0.88
	YW	WB4303	8179	-0.46	0.43	52627	-2.97	0.96
		WB4458	12540	-0.7	0.34	29195	-1.65	0.79
		WB-Grainfield	17717	-1	0.71	52981	-2.99	0.9
		Zenda	6072	-0.34	0.38	39271	-2.22	0.85
	IPP	WB4303	11550	-0.65	0.32	42450	-2.4	0.94
		WB4458	6951	-0.39	0.18	22194	-1.25	0.87
		WB-Grainfield	14791	-0.83	0.48	42649	-2.41	0.99
		Zenda	6957	-0.39	0.24	40771	-2.3	0.94
2019	FP	WB4303	26758	-1.48	0.69	36625	-2.03	0.8
		WB4458	32012	-1.77	0.63	35855	-1.98	0.86
		WB-Grainfield	21322	-1.18	0.62	44299	-2.45	0.85
		Zenda	15898	-0.88	0.56	44927	-2.49	0.8
	EF	WB4303	16570	-0.91	0.62	44342	-2.45	0.87
		WB4458	33949	-1.88	0.71	47040	-2.6	0.82
		WB-Grainfield	17626	-0.97	0.6	50907	-2.82	0.88
		Zenda	9402	-0.52	0.66	46259	-2.56	0.8
	EI	WB4303	6785	-0.37	0.57	27703	-1.53	0.75
		WB4458	11064	-0.61	0.73	31988	-1.77	0.88
		WB-Grainfield	5855	-0.32	0.7	26264	-1.45	0.9
		Zenda	5462	-0.3	0.4	34998	-1.94	0.73

IFP	WB4303	7613	-0.42	0.59	23148	-1.28	0.73
	WB4458	7682	-0.42	0.59	26739	-1.48	0.95
	WB-Grainfield	8291	-0.45	0.5	19400	-1.07	0.9
	Zenda	3890	-0.21	0.84	17900	-0.99	0.78
Yw	WB4303	6364	-0.35	0.82	23117	-1.28	0.8
	WB4458	6263	-0.34	0.18	24592	-1.36	0.88
	WB-Grainfield	4936	-0.27	0.5	16776	-0.93	0.79
	Zenda	2421	-0.13	0.45	12398	-0.68	0.61
IPP	WB4303	-7010	0.39	0.33	11958	-0.66	0.5
	WB4458	-1835	0.11	0.06	13874	-0.76	0.42
	WB-Grainfield	-118	0.01	0	14414	-0.79	0.69
	Zenda	-3355	0.19	0.26	14344	-0.79	0.89
